# Future-oriented cognition: links to mental health problems and mental wellbeing in preschool-aged and primary-school-aged children

**DOI:** 10.3389/fpsyg.2023.1211986

**Published:** 2023-09-27

**Authors:** Jessica Marks, Silvia Schneider, Babett Voigt

**Affiliations:** ^1^Mental Health Research and Treatment Center, Ruhr University, Bochum, Germany; ^2^German Center for Mental Health (DZPG), partner site Bochum/Marburg, Germany

**Keywords:** future-oriented cognition, pre-schoolers, primary-schoolers, mental health-problems, mental wellbeing

## Abstract

Future-oriented cognition plays a manifold role for adults’ mental health. The present study aimed to investigate the relationship between future-oriented cognition and mental health in *N* = 191 children aged between 3 and 7 years. Parents completed an online-questionnaire including children’s future-oriented cognition (e.g., episodic foresight; Children Future Thinking Questionnaire; CFTQ), children’s mental health problems (Strengths and Difficulties Questionnaire; SDQ), and wellbeing (Parent-rated Life Orientation Test of children; PLOT and Positive-Mental-Health Scale; PMH). More externalizing problems (especially hyperactivity) related to lower future-oriented cognition. For mental wellbeing, higher levels of optimism were associated with higher episodic foresight. Future-oriented cognition increased with age cross-sectionally. This increase was flatter at higher levels of wellbeing (indicated by lower pessimism). Results are discussed considering findings on the role of future-oriented cognition for mental health in adults and adolescents. Suggestions for future work are presented regarding the direction of the observed links and underlying mechanisms.

## Introduction

One of the most exciting human abilities is to think about one’s personal future going beyond the here-and-now and beyond one’s past experiences. The ability to look into one’s possible future helps humans to make decisions, to pursue personal goals, to regulate emotions and thus contributes to adaptive functioning and wellbeing in manifold ways. In turn, dysfunctional modes of future thinking can have manifold negative consequences and thus pose a threat to mental health. Consequently, the emergence of future thinking in childhood may represent opportunity as well risk. Along both routes, interindividual differences as well as differences in the development of future thinking may relate to children’s mental health. A possibility that received little systematic empirical investigation so far. The present work aims to fill this gap.

First signs of mental health problems are observable already in infancy and toddlerhood (Mäntymaa et al., 2012) with symptoms like being clingy or regulatory problems (Degangi et al., 1993; Schiele and Domschke, 2021). Such early psychopathological symptoms do not seem to simply go away, but might persist, cumulate and form a poor starting point for further development (e.g., Hemmi et al., 2011). These psychopathological problems can be divided into two areas that are both diagnostically (American Psychiatric Association and American Psychiatric Association, 2013) and empirically (Cicchetti and Toth, 1992) validated: internalizing and externalizing mental health problems. These terms are used to describe two broad-band groupings of behavioral, social and emotional problems (Achenbach et al., 2016).

In turn, mental wellbeing is considered as a resilience factor that protects children against disadvantageous developmental trajectories. Mental wellbeing is associated with better physical health and longevity and, thus, has implications for life-span health (Steptoe et al., 2015; Trudel-Fitzgerald et al., 2019). Going beyond the mere absence of psychopathology, mental wellbeing encompasses high positive affect, including high optimism, low negative affect and high life satisfaction (subjective wellbeing; Diener, 1984; Diener et al., 1985) as well as autonomy, self-acceptance, environmental mastery, purpose in life, positive relationships with others, and personal growth (psychological wellbeing; Ryff and Singer, 1996). As mental wellbeing and mental health problems are seen as independent but related concepts (Keyes et al., 2002; Schönfeld et al., 2016), finding out more about the correlates and mechanisms of early mental health is of critical importance and should consider both concepts (Suldo and Shaffer, 2008).

In the search of risk and protective factors for early mental health, the role of specific cognitions has been widely neglected compared to the rich literature on the role of temperamental reactivity and parental behavior (e.g., Sroufe, 2013). Future-oriented cognition may be of particular interest in this regard, as manifold findings from research with adults suggests (Suddendorf, 2017). Future-orientated cognition comprises of several facets. While a debate about a common taxonomy is ongoing, the following components have been in the focus of previous research (e.g., Szpunar et al., 2014): episodic foresight/episodic future thinking (ability to mentally project oneself into a future situation; Atance and Neill, 2001), delay of gratification (voluntary postponement of immediate gratification for the sake of greater future gains; Mischel et al., 1989), saving behavior (ability, to reserve resources in the present for the sake of future enjoyment; Metcalf and Atance, 2011), prospective memory (ability to remember to carry out future intentions; Kliegel and Theodor, 2007), planning (constructing plans/goals and envisioning the actions necessary to achieve those future goals; Shapiro and Hudson, 2004). Current research has particularly focused on episodic foresight/episodic future thinking (EFT) because it appears to have explanatory value for the other future-oriented facets of cognition and behavior (for a review: Atance and O’Neill, 2005). Developmental literature showed that the preschool period (3–5 years) represents a critical time window for qualitative and quantitative changes in future-oriented cognition (e.g., Atance and O’Neill, 2005; Atance and Meltzoff, 2006; Suddendorf and Redshaw, 2013) and that future-oriented cognition may become more and more important in mastering increasing external demands to self-regulate behavior and emotions autonomously from preschool age to primary school age (e.g., Bufferd et al., 2016).

In general, researchers widely agree that facets of future-orientated cognition play a critical role for psychological functioning in adults (MacLeod, 2016; for an overview see Bulley et al., 2018). On the one hand, they provide considerable adaptive value (e.g., Suddendorf, 2017) supporting important psychological functions such as decision making and emotion regulation (see Schacter et al., 2017 for a review). On the other hand, altered future-oriented cognition can have unfavorable effects for mental health, as numerous studies have documented in the last few decades (e.g., Roepke and Seligman, 2016). Whether mental health already in childhood is related to interindividual differences in future-oriented cognition remains unclear and may depend on the child’s age, external demands, and developmental tasks that the child is expected to master at a given age. Thus, following the models of developmental psychopathology, it is also of interest whether mental health relates to different age-related patterns (e.g., Rutter and Sroufe, 2000; Cummings and Valentino, 2015). However, so far it remains open whether higher vs. lower levels of mental health relate to a decelerated (flatter increase with age) or an accelerated (steeper increase with age) age-related pattern of future-oriented cognition.

### Internalizing problems and future-oriented cognition

The way a person thinks about and behaves with respect to the future is often described as the central feature of anxiety disorders and depression (Miloyan et al., 2014; for a review). Accordingly, manifold correlational studies with adults and adolescents have related internalizing symptoms such as worrying or depressive symptoms with altered future-oriented cognition and expectations about future events (MacLeod and Conway, 2005; Schubert et al., 2020; Hallford et al., 2020a). Specifically, compared to healthy control subjects, depressed adults reported less frequent mental imagery, reported less specific and plausible simulations, with less anticipatory pleasure and a more pessimistic view of the future (Miranda and Mennin, 2007; Miloyan et al., 2014; Holmes et al., 2016; Hallford et al., 2020a). Individuals with generalized anxiety disorders showed less spontaneously generated simulations of future events with less detail and perceived negative future events as more plausible than a healthy control group (Wu et al., 2015). Experimental studies confirmed these findings and suggest that the observed link may provide possible implications for clinical practice (e.g., Renner et al., 2021). For example, training or instructing EFT about positive future events did not only elicit positive affect in the here-and-now, but also increased anticipated pleasure and the intention to perform related activities in healthy controls (Hallford et al., 2020a; Westermann et al., 2021; Hallford et al., 2022). Beyond EFT, there is some, albeit considerably less evidence, that also other facets of future-oriented cognition such as delay discounting, planning and prospective memory may be altered in subjects showing internalizing symptoms (Miloyan et al., 2014; McFarland and Vasterling, 2018; for a review). However, in contrast to the fast growing body of literature on adults, analogue investigations in children, and particularly in young children before school age, are still scarce (Visu-Petra and Opriş, 2019). School-aged children with internalizing symptoms have been found to show alterations in cognition (Epkins, 2000) as well as deficits in neurocognitive skills critical for EFT (e.g., executive functions, memory, verbal fluency; Suddendorf and Redshaw, 2013; Blanken et al., 2017; Nelson et al., 2018) as well as planning and prospective memory (Mahy et al., 2014). As one of the few direct investigations in younger children, Opriş et al. (2021) found that preschool-aged children (aged 3–6 years) with higher trait anxiety performed poorer in an item-choice EFT task. Taken together, correlational and experimental evidence suggest that altered future-oriented cognition (particularly EFT) may play a role in the context of internalizing problems in adults. Further, some preliminary evidence suggests that this may be also true for children, even before school-age. However, more research replicating and extending this early finding is highly warranted. Such research should also consider possible links to other facets of future-oriented cognition apart from EFT and to the age-related patterns of these facets.

### Externalizing problems and future-oriented cognition

Past research has well documented that some externalizing symptoms such as impulsivity, hyperactivity and inattention can manifest themselves in low skills in delaying gratification or difficulties in organizing behavioral steps in order to achieve a goal, that is, they become evident in reduced future-oriented cognition (e.g., Bromberg et al., 2015; Patros et al., 2019). For example, adolescents with higher levels of reactive aggression showed lower delay of gratification skills and more difficulties in projecting themselves into distant future scenarios, compared to adolescents with less reactive aggression (Ahmed et al., 2018). In addition, school-aged children with ADHD had lower planning skills compared to peers without ADHD (Patros et al., 2019; for a review). Those results have been confirmed by experimental and longitudinal studies. For example, a training in forming if-then plans (Gawrilow et al., 2011) reduced deficits in delay gratification in children with ADHD, suggesting that planning may also play a role in the context of externalizing symptoms. In accordance with this, school-aged children (6–15 years) showing more externalizing problems in the Child Behaviour Check List (CBCL) showed fewer developmental gains in planning over time (Fuhrmann et al., 2022). Beyond the link of externalizing problems with delay of gratification and planning, the role of future-oriented cognition in general and other facets such as EFT remain widely neglected particularly in studies on young children.

### Mental wellbeing and future-oriented cognition

Humans think a great deal about emotionally significant events of their personal future (D’Argembeau et al., 2011; for an overview), which elicits respective affects in the here-and-now (Schubert et al., 2020; for a review). This may not only trigger negative affect that is examined in the context of internalizing symptoms, but also positive affect that has been studied in the context of mental wellbeing (Seligman et al., 2005; Springer et al., 2011; Andrews-Hanna et al., 2013). Indeed, various studies have reported a link between positive future thinking and mental wellbeing (Schmuck and Sheldon, 2001; for an overview; MacLeod and Conway, 2007; MacLeod, 2016 for a review). For example, when thinking about the future, subjects showing higher levels of optimism (i.e., subjective wellbeing; Diener, 1984; Diener et al., 1985) were more likely to report positive mental imagery and lower negative mood (Ji et al., 2022; for example). In addition to correlational evidence, experimental studies examined how wellbeing could improve by manipulating adults’ future-oriented cognition (Meevissen et al., 2011; Jing et al., 2016; Busby Grant and Wilson, 2021) with positive effects on optimism (Meevissen et al., 2011; Peters et al., 2013) and more global measures of mental wellbeing (Lyubomirsky et al., 2011; Lopes et al., 2016). For example, Holmes et al. (2009) showed that imagining a positive event increased positive mood and even protected against later negative mood induction. Furthermore, over a two-week period, Quoidbach et al. (2009) instructed participants to imagine in detail four positive events that could reasonably happen to them in the near future. Subjects’ happiness increased within these 2 weeks compared to participants who had been instructed to image negative and or neutral future events (see Gamble et al., 2021; for similar results on positive affect). In addition, Jing et al. (2016) showed that increasing the episodic detail via a specificity induction for a future behavior seems to enhance psychological wellbeing. So, empirical literature clearly supports a relation between different facets of future-oriented cognition and mental wellbeing in adults. However, research about mental wellbeing of (young) children is still in its early stages (Domínguez-Serrano et al., 2019), and explicit investigations of a possible link to different facets of future-oriented cognition and age-related patterns is lacking so far.

### The present study

In summary, future-oriented cognitions were shown to be a critical factor for adults’ mental health, including mental health problems as well as mental wellbeing in a great variety of studies (e.g., Schmuck and Sheldon, 2001; Miloyan et al., 2014; Ahmed et al., 2018; Schubert et al., 2020; Forster et al., 2021; Ji et al., 2022). Respective research is rare for preschool-age, although preschool-age is marked by considerable developmental steps with respect to future-oriented cognition (McCormack and Hoerl, 2020; for a review). Given the fact that mental problems already manifest themselves during this period and set the stage for disadvantageous outcomes later in life, research on the link between mental health and future-oriented cognition is highly warranted. As a first step, the present study aimed to provide further detail into this relationship in a low-risk sample of children aged between 3 and 7 years, in a non-experimental, correlational study. In contrast to previous studies, we focused on early childhood, combined a developmental and a clinical perspective, considered mental health problems as well as positive mental health, and took into account different facets of future-oriented cognition.

We pursued two main aims. First, we wanted to investigate whether early indicators of parent-reported mental health in children aged 3–7 years already relate to individual differences in future-oriented cognition based on the observation by their parents in everyday life, as suggested by findings in adult and adolescent literature (e.g., Ahmed et al., 2018; Hallford et al., 2020a). Second, relying on parent reports, we wanted to find out whether early indicators of mental health would be associated with different cross-sectional age-related patterns of future-oriented cognitions, as suggested by models of developmental psychopathology (Rutter and Sroufe, 2000; Hemmi et al., 2011). Based on the findings from the adult and adolescent literature, we assumed a generally protective role of future-oriented cognition. In detail, we first hypothesized that children with less mental health problems and higher levels of wellbeing would show more future-oriented cognition, compared to children with more mental health-problems and lower levels of mental wellbeing (MacLeod, 2016; for an overview see Bulley et al., 2018). Further, we expected that future-oriented cognition would show a less steep increase across age cross-sectionally when the number of mental health problems would be higher (decelerated age-related pattern), as suggested by findings from Fuhrmann et al. (2022), based on models of developmental psychopathology (Rutter and Sroufe, 2000; Hemmi et al., 2011; Schönfeld et al., 2016). Finally, we predicted that future-oriented cognition would show a steeper increase across age cross-sectionally when the level of mental wellbeing would be higher (accelerated age-related pattern; this project was preregistered at OSF; https://osf.io/8mcdq).

The specific nature of the relations of future-oriented cognition seems to vary with the domain of mental health problems and the facet of future-oriented thinking. Consequently, we also explored relations distinguishing between internalizing and externalizing problems (Cicchetti and Toth, 1992; American Psychiatric Association and American Psychiatric Association, 2013) and different facets of future-oriented cognition (Szpunar et al., 2014). This distinction was likely to give further insights as externalizing problems have been associated with lowered future-oriented cognition (e.g., Ahmed et al., 2018), whereas more future-oriented thinking had been linked to internalizing problems when it was negatively biased (e.g., Roepke and Seligman, 2016). However, we refrained from explicitly formulating *a priori* hypotheses for each domain and facet given the sparse research with (preschool-aged) children.

## Methods

### Participants

The final sample comprised of *N* = 191 caregivers who reported about their child (58.6% female, 39.8% male and 1.6% diverse) using the Qualtrics software (Version 07/2020). Children were between 3 or 7 years (3.0–7.11) with an average age of *M* = 5.68 years (*SD* = 1.29). Children’s parents were predominantly German (85.3% of the fathers and 91.6% of the mothers) and many caregivers held a college degree (65.2% of the fathers and 74.3% of the mothers). Recruitment for the online survey took place via social media and various parent/caregiver websites. Participants could take part in a voucher lottery for 5×10 Euro vouchers.

Using G*Power (Faul et al., 2009), it was estimated to collect valid data from about 250 participants in a period of about 1 year starting in July 2020. That would have been sufficient to detect even small effects of *f^2^* = 0.03 given a statistical power of 0.80 and an alpha level of *α = 0*.05 (Cohen, 1992). Recruiting was stopped after the planned survey period ended. At this time *n* = 324 caregivers had opened the questionnaire overall. As we included only data of those parents who at least completed the CFTQ and the SDQ, *n* = 133 of them provided incomplete answers and were excluded from analyses. The final sample size of *N* = 191 valid data sets was still large enough to detect small effects of *f^2^* = 0.03 given a statistical power of 0.80 and an alpha level of *α* = 0.05. The study was ethically approved by the Ethics Committee of the faculty for psychology at the Ruhr-University Bochum. Informed consent was acquired before each parent administered the questionnaire online.

### Measurements

#### Children’s future thinking questionnaire

Future-oriented cognition was assessed using the Children’s Future Thinking Questionnaire (CFTQ; Mazachowsky and Mahy, 2020). The CFTQ is a parent-report measure for assessing future-oriented cognition in children between 3 and 7 years. It comprises of 44 items that belong to one of five scales: Saving, Prospective Memory Items, Episodic Foresight, Planning and Delay of Gratification (see Table 1). Items are formulated as statements about children’s ability of the respective facet in everyday life [e.g., “Thinks about what might be needed for future excursions (e.g., bringing toys/ books on a long car ride)”]. Parents were asked to indicate their agreement to the respective statement using a six-point scale ranging from *strongly disagree* (1) to *strongly agree* (6) including the options to answer *do not know* (7), *does not apply* (8), *prefer not to answer* (9). Subscales scores were created based on the mean of the respective items. The average across all five subscale scores served as CFTQ total score. On all scales, higher values indicated higher future-oriented cognition. Previous studies have documented the high internal consistency, strong reliability and excellent test–retest reliability of the CFTQ (Mazachowsky and Mahy, 2020). We present results for the CFTQ total score as Mazachowsky and Mahy (2020) suggested that the five subscales seem to converge into one single factor. We additionally explored findings for the CFTQ subscales with a special focus on EFT given the critical role of EFT for mental health, the rich developmental literature about this facet, and the fact that the EFT subscale related to respective behavioral tasks, indicating convergent validity (Mazachowsky and Mahy, 2020). For the present purposes, the questionnaire was translated into German in a standardized multi-step procedure suggested by current guidelines (Gudmundsson, 2009; e.g., qualified translators, forward translation, review of forward translation, back-translation, review of back-translation, adapting items and documentary of adaption, repetition of these steps in a loop until the translation is decent). In the present sample, the internal consistency for CFTQ in total proved to be good with *α* = 0.86 (Ferketich, 1990). The internal consistency for scales ranged between *α* = 0.74 (delay of gratification) and *α* = 0.86 (total score). For interpretation, an alpha coefficient of at least 0.70 to be adequate for an instrument in early stages of development and a coefficient of at least 0.80 to be adequate for a more developed instrument (Ferketich, 1990).

**Table 1:** Presents descriptive statistical values for the total scores and subscale scores of the CFTQ, SDQ, PMH, and PLOT.

		*M*	*SD*	Range		
CFTQ						
	Total score	4.21	0.59	2.57–5.86		
	Episodic foresight	4.28	0.73	2.33–6.00		
	Saving	4.17	0.63	2.63–5.78		
	Prospective memory	4.43	0.75	2.50–6.00		
	Planning	4.39	0.77	2.00–6.00		
	Delay of gratification	3.97	0.74	2.00–5.78		
SDQ						
					Cut-off^a^	% over cut-off^a^
	Total score	8.37	4.87	0.00–24.00	18–40	6%
	Emotional problem	1.86	1.91	0.00–9.00	7–10	12%
	Hyperactivity	3.19	2.15	0.00–9.00	8–10	7%
	Conduct problem	2.13	1.60	0.00–7.00	5–10	19%
PMH						
	Total score	3.53	0.36	2.22–4.00		
PLOT						
	Total score	4.12	0.49	2.00–5.00		
	Optimism	3.67	0.62	1.50–5.00		
	Pessimism	4.57	0.55	2.00–5.00		

*N* = 191; CFTQ, Children’s Future Thinking Questionnaire and subscales; SDQ, Strengths and Difficulties Questionnaire and Subscales; PMH, Positive Mental Health Scale; PLOT, Parent-rated Life Orientation Test of Children. ^a^Cut-off points for abnormal scores and percentage for children above the cut-off; for more information see https://www.sdqinfo.org/a0.html.

#### Strengths and difficulties questionnaire

The German parent-version of the Strengths and Difficulties Questionnaire (SDQ; Goodman, 1997) was used to ascertain mental health problems (version for 2–4 year-olds or version for 4–17-years depending on the child’s age). The German version of the SDQ is a widely used mental health screening that is well-validated (Klasen et al., 2003), contains 25 items and has five scales: Emotional Problem Scale, Conduct Problem Scale, Hyperactivity Scale, Peer Problems Scale and Prosocial Scale. In addition to the sum score for each subscale, the SDQ total score can be computed as a general measure of mental health problems by summing the scores for all subscales (except the Prosocial Scale). For the present purposes, the Emotional Problem Scale served as an indicator for internalizing problems. Externalizing problems were measured in terms of the Conduct Problem Scale and the Hyperactivity Scale (Goodman et al., 2010; Caci et al., 2015). Parents rated the behavior of their child in the last 6 months (e.g., “Often has temper tantrums or hot tempers.”) on a scale ranging from *not true* (0) to *certainly true* (2). Higher scores on the SDQ scales indicated a greater risk of mental health problems (Koglin et al., 2007). Based on the available norms, it is possible to identify marginal or remarkable values indicating heightened risk for mental health problems (Koglin et al., 2007). For this work, the classification of values is based on previous work with German subjects showing normative data (Woerner et al., 2002; Klasen et al., 2003). In previous studies, the SDQ showed good test–retest reliability and validity as indicated by convergence with other questionnaires and diagnostic test for psychopathology in children (e.g., CBCL; Klasen et al., 2003). The internal consistency of the SDQ total score for this study proved to be acceptable with *α* = 0.66 (Ferketich, 1990). The internal consistency for scales ranged between *α* = 0.55 (Conduct Problems Scale) and *α* = 0.78 (Hyperactivity Scale). The Conduct Problems subscale of the SDQ has an *α* = 0.55, and is therefore questionable (Blanz, 2015). Similar observations have been reported by past studies (e.g., Woerner et al., 2004). We inspected the inter-item correlations. Nearly all items formed significant bivariate correlations (*rs* ≥ 0.16, *ps* ≤ 0.009), except for item 7 which did not relate to item 12 (*r* = 0.13, *p* = 0.077) and item 22 (*r* = 0.14, *p* = 0.059). Forming a subscale without item 7 yielded in a slightly improved Cronbach’s Alpha of *α* = 0.59, which is similar to previous findings for the German SDQ and the conduct-problem subscale (e.g., *α* = 0.60 in Woerner et al., 2004). Item 7 (‘My child is obedient’) stands out from the subscale in terms of content compared to the other items, which are about tantrums, stealing, arguing, or lying. However, omitting item 7, did not change our findings (e.g., regarding the relation of SDQ subscale conduct problems and the CFTQ total score, *p* = 0.118). For this reason, we assume that the low Cronbach’s alpha value of 0.55 for the Conduct Problem Scale is not the reason for missing correlations.

#### Positive mental health scale

We ascertained mental wellbeing using a new parent-report measure that was based on the German Positive Mental Health Scale (*PMH-Scale*; Lukat et al., 2016). The PMH-Scale is a short, unidimensional measure of positive mental health which considers the presence of general emotional, psychological and social wellbeing (Keyes et al., 2002; Lukat et al., 2016). In adults, it turned out to be a reliable and internally consistent measure that shows convergent and discriminant validity (Lukat et al., 2016; Velten et al., 2022). We developed a parent-report measure for children by rephrasing the nine self-report items (e.g., “Much of what my child does brings him/her joy.”) while maintaining the answer-scale ranging from *do not agree* (0) to *agree* (3). Higher scores on the PMH-Scale indicated higher mental wellbeing of the child as rated by the caregiver. The internal consistency for this study proved to be good with α = 0.82 (Ferketich, 1990). Answers from one caregiver were missing for the PMH scale so that respective analyses based on data of *n* = 190.

#### Parent-rated life orientation test of children

The Parent-rated Life Orientation Test of children (*PLOT*; Lemola et al., 2010) was used to assess children’s disposition for optimism and pessimism as one component of wellbeing (Diener, 1984; Diener et al., 1985). Optimism and pessimism denote one’s generalized positive and negative expectations about the future (e.g., “When entering a new situation, my child expects to have fun”). The PLOT was developed based on the Life Orientation Test and its revisions (Scheier and Carver, 1985; Scheier et al., 1994), a standard instrument for measuring dispositional levels of optimism in adults. The PLOT has eight items, four for ascertaining optimism and four for pessimism. Parents indicated their agreement on a 5-point scale ranging from *does not apply at all* (1) to *totally applies* (5). We present findings for both, the PLOT total score and both subscales as Lemola et al. (2012) presented evidence for a better fit of a two-factor solution. The literature is increasingly coming to the conclusion that pessimism and optimism are two different constructs and are not polar opposites (Chang et al., 1994; Scheier et al., 1994; Kivimäki et al., 2005). Higher scores on the PLOT total score and the optimism subscale indicated higher expectations of positive experiences and higher scores on the pessimism subscale indicated lower expectations of negative events. In previous studies, the PLOT showed good reliability, convergent and discriminant validity (Lemola et al., 2012). The internal consistency for this study also proved to be good (Ferketich, 1990) with α = 0.78. Answers from five caregivers were missing for the PLOT so that respective analyses based on *n* = 186.

### Procedure

At the beginning of the online questionnaire, parents were informed about the goals and the background of the study. In addition, information was given about the anonymity of data collection and the voluntary nature of participation. After questions about sociodemographic characteristics, parents filled in the CFTQ (future-oriented cognition), then the SDQ (mental health problems), the PMH and the PLOT (wellbeing).

### Planned statistical analyses

Preliminary analyses comprised of testing for age-related effects (bivariate correlations) and of testing for gender-related effects (*t*-tests). We also planned to conduct bivariate correlations for testing relations among indicators of mental health problems (SDQ and subscales), among indicators of mental wellbeing (PMH, PLOT and subscales), and between indicators of mental health problems and wellbeing. For testing H_1a-b_ (mental health problems) and H_2a-b_ (mental wellbeing), we planned two separate linear regression analyses with future-oriented cognitions CFTQ as dependent variable. In the first analysis testing H_1a-b_ (mental health problems), we entered age, gender, the measures of mental health problems (SDQ total score and subscales) and the interaction between age and mental health problems (SDQ total score and subscales) as predictors in one step. In the second analysis testing H_2a-b_ (mental wellbeing), we entered age, gender, the measures of mental wellbeing (PMH total score, PLOT total score and subscales) and the interaction between age and mental wellbeing (PMH total score, PLOT total score and subscales) as predictors in one step. Beta coefficients served as proxy for effect size. In case of a significant interaction, we planned to employ simple slope analyses, i.e., exploring the link between age and future-oriented cognition at high and low levels of mental health problems or mental wellbeing (+/− 1 SD) respectively (Preacher and Hayes, 2004). Statistical analyses were accomplished using IBM SPSS Statistics Version 27.0 (IBM Corp,. 2020).

## Results

### Descriptives

### Preliminary analyses: bivariate relations

Older children had higher CFTQ total scores [*r*(186) = 0.44, *p* < 0.001] and higher scores on all five subscales of the CFTQ (*ps* ≤ 0.001), replicating the age effect reported in past research (e.g., Hudson et al., 2011 for a review). Additionally, older children received lower SDQ-Conduct Problem scores [*r*(186) = −0.228, *p* = 0.002], but no further age-related effects were found. Respecting gender, girls (*M* = 4.31, *SD* = 0.57) showed significantly higher CFTQ total scores than boys (*M* = 4.09, *SD* = 0.61), *t*(186) = 2.53, *p* = 0.014. Similarly, girls showed higher scores for CFTQ Planning [girls: *M* = 4.54, *SD* = 0.71, boys: *M* = 4.19, *SD* = 0.82, *t*(186) = 3.05, *p* = 0.003] and higher scores for CFTQ EFT [girls: *M* = 4.22, *SD* = 0.73, boys: *M* = 3.92, *SD* = 0.75, *t*(186) = 2.66, *p* = 0.009] compared to boys. No further gender-related effects emerged. Based on these preliminary analyses, we decided to consider age and gender in each of the planned analyses. The CFTQ was approximately normally distributed (*p* > 0.05), but SDQ, PMH and PLOT (*p* < 0.05) were not, as the Shapiro–Wilk-Test revealed (Shapiro and Wilk, 1965). For the SDQ, distribution skewed to the right, for the PMH and PLOT, distribution skewed to the left. Due to the deviations from normal distribution, all analyses were carried out using the robust bootstrapping method requesting 1,000 bootstrap samples and using simple resampling (Rasmussen, 1987).

Next, we inspected the relations among mental health measures. We found evidence for conceptual validity of the parent-report version of the PMH scale for children. A positive correlation with the PLOT, indicated convergent validity, *r*(186) = 0.60, *p* < 0.001. In addition, a negative correlation with the SDQ total score [*r*(186) = −0.65, *p* < 0.001] and the SDQ subscales (Hyperactivity Scale, Emotional Problem, Conduct Problem Scale) indicated discriminant validity, ranging from *r*(186) = −0.34 to −0.56, *ps* < 0.001, respectively. Also in accordance with the dual factor model of mental health, there was a moderate negative correlation between the SDQ total score and the PLOT, *r*(186) = −0.51, *p* < 0.001 (Suldo and Shaffer, 2008). For an overview of all correlations see Supplementary Table S1.

### Mental health problems and future-oriented cognition – hypothesis 1a, 1b

**Figure 1:**
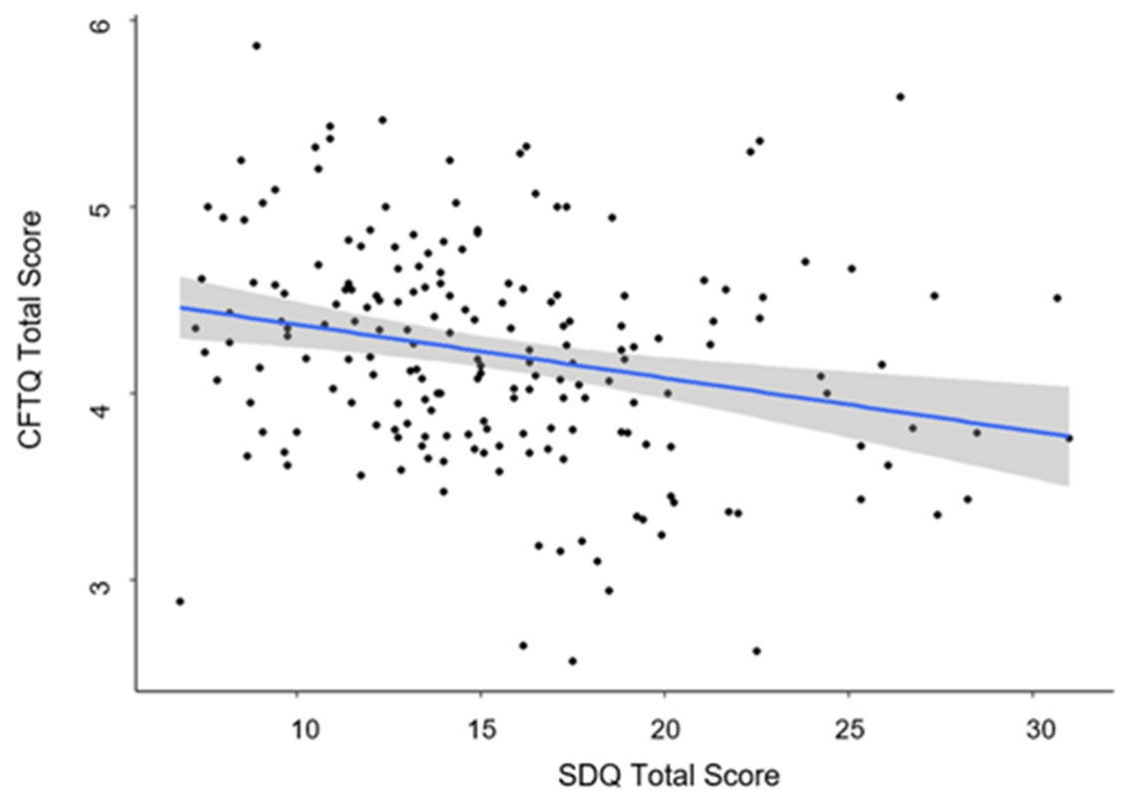
Scatterplot for the relation between mental health problems (SDQ total score) and future-orientated cognition (CFTQ total score). *N* = 190. Regression line and 95% confidence interval (CI) predicting future-oriented cognition (CFTQ total score) by the SDQ total score and accounting for age and gender.

**Figure 2:**
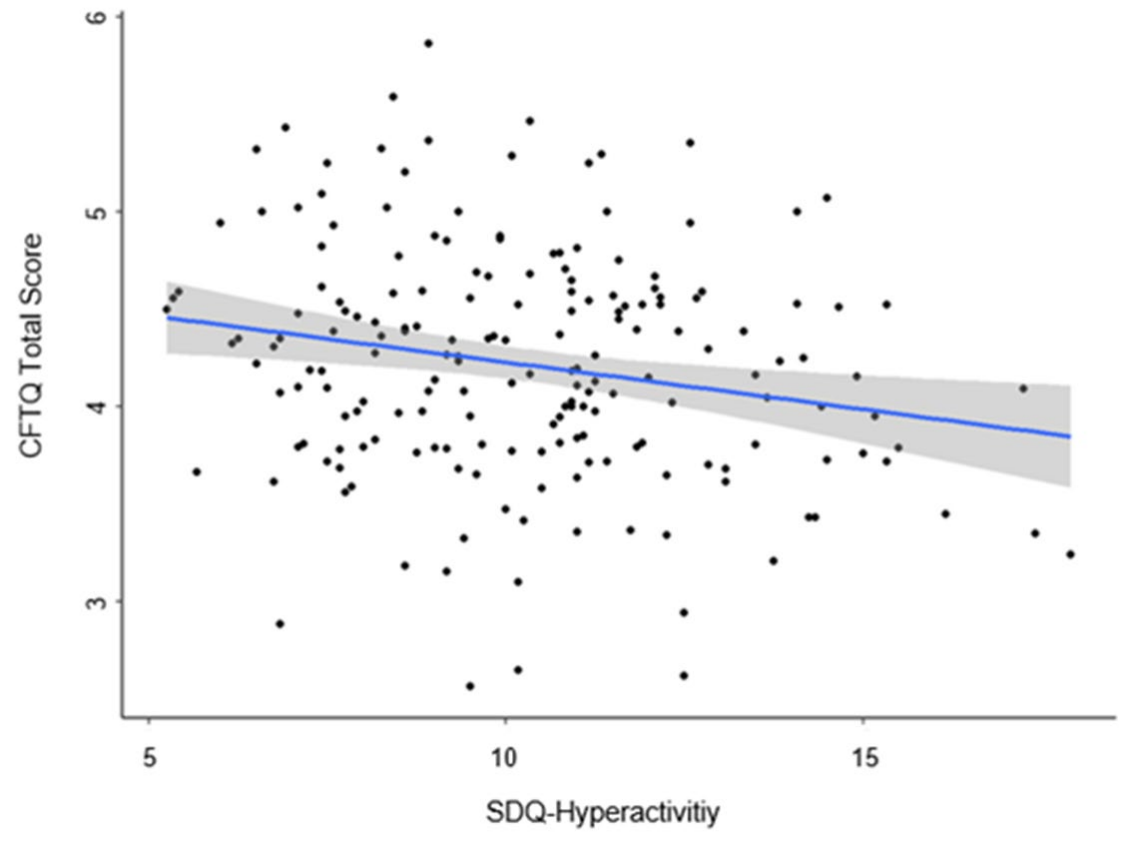
Scatterplot for the relation between hyperactivity (SDQ) and future-orientated cognition (CFTQ total score). *N* = 190. Regression line and 95% confidence interval (CI) predicting future-oriented cognition (CFTQ total score) by the SDQ-Hyperactivity and accounting for age and gender.

According to regression analyses, a higher SDQ total score predicted lower scores on the CFTQ [β = −0.23 *t*(184) = −3.64; *p* ≤ 0.001], even when considering age and gender at the same time. Thus, children with more mental health problems overall showed less future-oriented cognition in everyday life according to their parents (see Figure 1). Examining the subscales of the SDQ revealed that a higher hyperactivity score of the SDQ predicted lower CFTQ scores when accounting for age [β = −0.41, *t*(180) = −6.27; *p* ≤ 0.001; see Figure 2], whereas scores for conduct problems and emotional problems were unrelated. Thus, Hypothesis 1a was partly supported and findings suggest that this relation depends on the domain of mental health problems. For Hypothesis 1b, analyzing the relation of age-related patterns, we found no significant interaction between age and the total score of the SDQ (*p* = 0.094) or any of the SDQ subscales (*p*s ≥ 0.332), indicating that the age-related increase of CFTQ is independent of children’s mental health problems.

The CFTQ considers a variety of facets of future-oriented cognition including EFT, delay of gratification, planning, saving, and prospective memory (Mazachowsky and Mahy, 2020). Thus, we were interested to explore whether the relations reported for the CFTQ total score would also emerge for the subscales, that is, for the different facets of future-oriented cognition. Therefore, we repeated statistical analyses described above with the CFTQ subscales.

For mental health problems, higher SDQ total scores predicted lower scores on all CFTQ subscales (from *p* ≤ 0.001 to *p* = 0.006). This relation was specific for SDQ-Hyperactivity; higher SDQ-Hyperactivity predicted lower scores on all CFTQ subscales (*p* ≤ 0.001). No further relations were found for the subscales of the SDQ and other subscales of the CFTQ (*p*s ≥ 0.122). Analogue to the main analyses, we further explored whether mental health indicators moderated the possible link between age and CFTQ subscales. With respect to mental health problems (SDQ), we found no significant interaction between age and the total score of the SDQ (*p* ≥ 0.115) or any of the SDQ-subscales (*p*s ≥ 0.122), indicating that the age-related increase of CFTQ-subscales was independent of children’s mental health problems.

### Mental wellbeing and future-oriented cognition – hypothesis 2a, b

According to a regression analysis, neither the PMH score nor the total score of the PLOT predicted the CFTQ score when accounting for age and gender at the same time, *ps* > 0.077. We explored the pessimism and the optimism factor of the PLOT separately in addition to age, gender and the PMH score as suggested by the 2-factor-solution found in Lemola et al. (2012). Findings revealed that neither PLOT-Optimism nor PLOT-Pessimism related to the CFTQ total score, *ps* ≥ 0.053. Thus, for Hypothesis 2a we found no significant correlation, indicating that the CFTQ is independent of children’s mental wellbeing.

**Figure 3:**
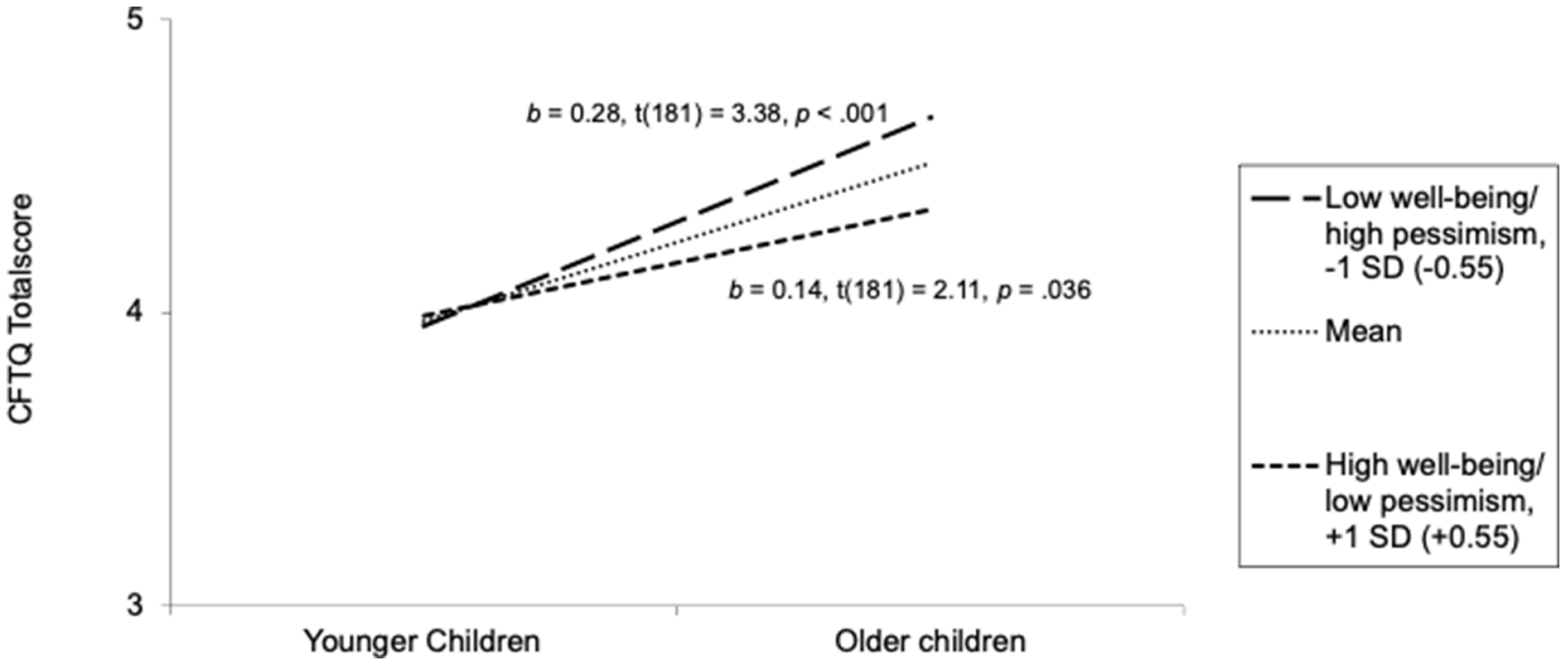
Relation between age and future-orientated cognitions (CFTQ) moderated by pessimism (PLOT). *N* = 185. Plotting of SSA of the relation between age and the CFTQ Total Score at low levels of PLOT-Pessimism (–1 SD) versus at high levels of PLOT-Pessimism (+1 SD). A higher PLOT-Pessimism score indicates lower expectations of negative events in the future, i.e., higher wellbeing.

Further, there was no interaction between age and the PMH or between age and the PLOT total score (*p*s ≥ 0.264), indicating that children’s mental wellbeing is not generally associated with age-related increases in the CFTQ. We then examined whether association between age and CFTQ depended on the PLOT subscales. According to the results, PLOT-Optimism did not moderate the relation between age and the CFTQ total score (*p* = 0.199), but there was a significant interaction term of age and PLOT-Pessimism (*p* = 0.023). Following the recommendations of Hayes (2018), the non-significant interaction term of the PLOT-Optimism was removed from the model, resulting in a final model with main effects of age and PLOT-Pessimism and a significant interaction term of age and PLOT-Pessimism [*β* = −0.132, *t*(181) = −2.03; *p* = 0.044]. To further check the interaction, we carried out a simple slope analysis (SSA) with +1 SD and –1 SD (see Figure 3). The corresponding graph revealed, that the CFTQ total score increases faster across age cross-sectionally at lower levels of mental wellbeing (lower values on the reversed PLOT-Pessimism subscale) compared to higher levels of mental wellbeing (higher values on the reversed PLOT-Pessimism subscale). Thus, hypothesis 2b was partly supported. Results suggest that this relation depends on the domain of wellbeing.

Respecting mental well-being and the subscales of the CFTQ, higher PLOT scores related to higher EFT scores [*β* = 0.139, *t*(177) = 2.08; *p* = 0.039]; specifically higher PLOT-Optimism related to higher EFT scores [*β* = 0.192, *t*(175) = 2.47; *p* = 0.015], when considering age and gender at the same time. No further relations with the other CFTQ subscales emerged for the PLOT (*p*s ≥ 0.081), and the PMH was not associated with any of the CFTQ subscales at all (*p*s ≥ 0.115).

Regarding age-related patterns of the CFTQ subscales, we found no interaction between age and the PMH or age and the PLOT or the PLOT-optimism (*p*s ≥ 0.273). However, there was a significant interaction between age and PLOT-Pessimism for the CFTQ-Prospective-Memory, CFTQ-Saving, and CFTQ-Planning (from *p* = 0.002 to *p* = 0.045). Accordingly, children with higher scores on the reversed PLOT pessimism scale (lower expectations of negative events indicating higher wellbeing) showed a flatter cross-sectional increase of prospective memory, saving as well as planning scores with age compared to children with lower pessimism scores (higher expectations of negative events indicating lower wellbeing). For an overview of all explorative analyses see Supplementary material.

## Discussion

An extensive body of research documents the manifold roles of future-oriented cognition with respect to mental health in adults. Moreover, during preschool-age critical advances in future-oriented thinking already occur (McCormack and Hoerl, 2020; for a review). Thus, the aim of the present work was two-folded. First, we investigated whether early indicators of mental health would relate to future-oriented cognition already in young children aged 3–7 years. Second, taking a developmental psychopathology perspective, we explored whether age-related differences in children’s future-oriented cognitions would vary among children with different levels of mental health. We thereby considered both mental health problems as well as mental wellbeing, based on the assumption that mental health is more than the absence of diseases (Keyes et al., 2002; Suldo and Shaffer, 2008; Schönfeld et al., 2016).

With respect to the first aim and in accordance with our hypotheses, we found that children with more mental health problems in general showed less signs of future-oriented cognitions in everyday life. This link emerged for hyperactivity (i.e., externalizing problems) in particular and for all facets of future-oriented cognition. However, there was no such relation to conduct problems and emotional problems (i.e., internalizing problems). For wellbeing, we found associations of future-oriented cognition with optimism and pessimism: In accordance with hypotheses, children rated to be more optimistic (i.e., a high general expectation of positive events, higher wellbeing) showed more signs of episodic foresight in everyday life, but not higher future-oriented cognition in general. With respect to the second aim, we found that older children showed more signs of future-oriented cognitions in everyday life (McCormack and Hoerl, 2020). However, this cross-sectional developmental pattern partly depended on children’s mental health. It was independent of mental health problems, but future-oriented cognition increased faster with age among more pessimistic children (i.e., a high general expectation of negative events, lower wellbeing). Explorative analyses indicated that this was particularly true for the cross-sectional development of prospective memory, saving and, planning. In turn, less pessimistic children (higher wellbeing) showed a slower cross-sectional development of future thinking and this was in direct contrast to our hypotheses.

The observed link between future-oriented cognition and externalizing problems (hyperactivity) is in line with research reporting a respective link in adults and adolescents (e.g., Rodriguez et al., 1989; Ahmed et al., 2018). The present study extends these results to preschool-aged children and other facets of future-oriented cognition such as episodic foresight. The observed relation to future-oriented cognition did not hold for conduct problems, however. This is in contrast to previous studies, showing for example a link between future-oriented cognition and reactive aggression in adolescents, aged 12–22 years (Ahmed et al., 2018). One possible explanation may be that some conduct problems such as lying, stealing, or spitefulness may not appear until later childhood. Another reason could be that parents may have underreported undesirable behavior such as tantrums or fights with other children. Observing respective behavior in the laboratory or involving further information sources such as clinical interviews or reports of kindergarten teachers may be helpful in this respect.

In contrast to externalizing problems, we did not find a respective relation for internalizing problems. This speaks against past research with adults (MacLeod and Conway, 2005; Miranda and Mennin, 2007; Miloyan et al., 2014; Schubert et al., 2020; Renner et al., 2021) and the finding that pre-schoolers with high trait anxiety show lower EFT performance in a behavioral task in the laboratory (Visu-Petra and Opriş, 2019). Several reasons may account for this finding. First, the CFTQ assesses the global quantity of future-oriented cognitions in terms of behavior in everyday life. However, it does not cover the content or the phenomenology of specific future-oriented thoughts (e.g., D’Argembeau et al., 2011) which have turned out to be particularly relevant for internalizing problems (e.g., Miloyan et al., 2014; for a review; Visu-Petra and Opriş, 2019). For example, symptoms of anxiety have been related to the valence and the perceived likelihood of future events. Furthermore, depression has been linked to lowered vividness of imagined positive upcoming events, as well as alterations in predicting positive affect in the future (Miranda and Mennin, 2007; Holmes et al., 2016; Miloyan et al., 2017; Hallford et al., 2020a,b). Therefore, deeper insights into the relation to internalizing problems require future studies that also consider the content and the phenomenology of future-oriented thoughts of young children.

Alternatively, the relation to internalizing problems may emerge after the age of 7 years, and children examined in this study may have been simply too young for the respective link. Accordingly, previous studies have shown that externalizing problems gradually decrease and internalizing problems gradually increase over age (Gilliom and Shaw, 2004) and that externalizing problems may be easier to observe for caregivers (Peacock et al., 2009). In line with this argument, internalizing problems (indicated by the emotional problem scale of the SDQ) were independent of age in the present sample. Therefore, future studies should consider a broader age range. Moreover, parents are close to the child and know the child well across manifold situations, speaking in favor for the use of parent ratings to assess mental health as was done in the present study. Future studies may gain new insights by combining parent-reports with (newly developed) self-reports and objective behavioral and physiological indicators of internalizing problems.

The observed relation between mental wellbeing with higher EFT in the present study is in line with manifold findings from adult research (Schmuck and Sheldon, 2001; Holmes et al., 2009; Meevissen et al., 2011; MacLeod, 2016; Schubert et al., 2020; Busby Grant and Wilson, 2021; Ji et al., 2022). It underscores a possible protective role of EFT for children’s development. In direct contrast stands our finding that higher wellbeing (less pessimism) is associated with a more decelerated development cross-sectionally. Thus, the (cross-sectional) development of future-oriented cognitions may be altered at lower levels of mental health as suggested by general frameworks of developmental psychopathology. However, the direction of the observed link seems surprising at the first glance in the light of past research. For example, Fuhrmann et al. (2022) showed that higher improvements of planning (collected over four age-waves) went along with fewer externalizing symptoms in childhood in school-aged children (higher levels of mental health), indicating that future-oriented cognition may function as a protective factor in childhood in accordance with findings from adult research (for an overview; Schmuck and Sheldon, 2001; MacLeod and Conway, 2005). One interpretation of the present results is that a more protracted development of future-oriented cognition may protect children against high levels of pessimism and thus benefit wellbeing, even though the present correlative design does not allow for conclusions about the direction of the observed link. This possible explanation points to the importance of considering the content and valence of future-oriented thoughts rather than merely how often future-oriented behavior occurs in general. The emergence of future thinking in childhood may not only represent opportunity when it entails positive thoughts about the future (as suggested by our findings for optimism), but it may also represent a risk when it entails negative thoughts about the future relatively early in ontogeny. Corroborating with this idea, previous studies found that higher levels of internalizing problems went along with more frequent negative future-oriented thoughts, but not more frequent future-oriented thinking in general (e.g., Roepke and Seligman, 2016; Hallford et al., 2020a,b). Thus, future studies with children should investigate whether and how the content of future-oriented thoughts may alter with age (e.g., in terms of a growing number of sorrows and fears). This approach would also help to gain a deeper understanding of the mechanisms that drive the link to optimism and the missing association with internalizing behavior problems. Beyond optimism and pessimism, no association emerged for the more global measure of wellbeing, the PMH-scale. One helpful next step to further explore this issue in more depth may be to combine parent-reports with (newly developed) self-reports and behavioral indicators of wellbeing for young children.

The present work was the first to show the correlation between future-oriented cognition and mental health in children as young as 3–7 years, considering multiple facets of future-oriented cognition and both aspects of mental health. This is an important first step in identifying future-oriented cognition as a critical factor for children’s mental health. However, different limitations of the present study make it necessary to replicate and further explore the present findings in a second step.

One limitation of the present study results from using convenience sampling what may have led to a quite homogenous sample with regard to socioeconomic background, culture and prevalence of clinically significant mental health problems. Participants mainly held a university degree (74% of the mothers), and were German (92% of the mothers). For example, socioeconomic advantage has been associated with higher cognitive skills in general and with a more far-sighted decision making so that our findings cannot be generalized to children coming from socioeconomic disadvantaged backgrounds (e.g., Letourneau et al., 2013). In addition, the range of mental health problems among the children was quite low what may have reduced our chances to find correlations. Future studies with risk samples may find higher and other correlations, e.g., such as a correlation to internalizing problems. In turn, relying on parent-report measures only raised the common method variance (CMV) and may have inflated the correlations. Another limitation is that the present correlative design does not allow for conclusions about the direction of the observed links. Furthermore, the fact that all measures are self-report by parents about their children may issue, especially since the study is correlational.

Therefore, future studies using multi-method approaches and experimental and/or longitudinal designs are required to replicate and explore the association between mental health and future-oriented cognition. In this context, at least two hypotheses aim to explain the role of cognitions for mental health in general. First, according to the *interference hypothesis* (Stawski et al., 2006), psychological distress often occurs in the context of psychopathological phenomena and disrupts cognitive processes. From this perspective, mental health problems function as risk factor for the development of future-oriented cognitions, whereas wellbeing is considered as a possible protective factor. For example, supporting the interference hypothesis, Okano et al. (2020) showed in a longitudinal study in children from third to ninth grade that externalizing symptoms aggravate academic achievements and cognitive development. Other studies also suggest that mental health problems precede cognitive development problems. For the planning facet of future-oriented cognition, Fuhrmann et al. (2022) were able to show that more externalizing symptoms in childhood were associated with less favorable planning trajectories.

In turn, the *cognitive reserve hypothesis* (Barnett et al., 2006) postulates that good cognitive functioning protects children against the emergence of mental health problems and supports wellbeing by allowing children to deal with stressful situations effectively. From this perspective, low levels of future-oriented cognitions are seen as one important source for low levels of mental health rather than a consequence of psychopathological processes. Supporting the cognitive reserve hypothesis, instructing participants to imagine a future event vividly led to a significant reduction in delay discounting, impulsive behavior and substance consumption (Peters and Büchel, 2010; Forster et al., 2021). Similar effects have been found for internalizing behavior such as depression and anxiety (Jing et al., 2016; Westermann et al., 2021).

Thus, empirical evidence exists for both accounts pointing to a possible bidirectional influence. This is in accordance with a third perspective provided by developmental psychopathology. It suggests that mental health and cognitive development are closely interwoven, thus, they interact bi-directionally over time leading to developmental cascades (e.g., Masten et al., 2005). Consequently, future longitudinal studies should investigate these processes over time. Furthermore, future experimental studies should investigate the mechanisms underlying the relation between mental health and future-oriented cognition in children (e.g., by affecting emotions, emotion regulation or the ability to meet daily external demands). The present findings suggest that the answer to those questions may differ for internalizing in contrast to externalizing problems and wellbeing, as well as for different facets of future-oriented cognition.

All in all, the present study provided early evidence for a connection between future-oriented cognition and mental health in children aged 3–7 years. Findings speak for a respective link to externalizing problems and hyperactivity, as well as wellbeing, optimism and pessimism. Evidence for an association to internalizing problems was not found. Future studies using multi-method, longitudinal and experimental approaches should further inspect the direction and the mechanisms of the link between future-oriented cognition, should consider different facets of mental health as well as the content and the phenomenology of future-oriented thoughts in samples covering a broader range of age and mental health problems. The present findings suggest that respective work would provide important implications for developmental psychopathological research and clinical practice.

## Data availability statement

The raw data supporting the conclusions of this article will be made available by the authors, without undue reservation.

## Ethics statement

The studies involving humans were approved by Ethic Committee of Ruhr-University Bochum (17.06.2020, No. 636). The studies were conducted in accordance with the local legislation and institutional requirements. The participants provided their written informed consent to participate in this study.

## Author contributions

JM performed the material preparation, data collection, and analysis. JM and BV wrote the first draft of the manuscript. All authors contributed to the study conception and design, commented on previous versions of the manuscript, and read and approved the final manuscript.

## Funding

This research was supported by a grant from the German Research Foundation (DFG; VO 23 25 / 2-1; AOBJ 65 81 82). Moreover we acknowledge support by the Open Access Publication Funds of the Ruhr-Universität Bochum.

## Conflict of interest

The authors declare that the research was conducted in the absence of any commercial or financial relationships that could be construed as a potential conflict of interest.

## Publisher’s note

All claims expressed in this article are solely those of the authors and do not necessarily represent those of their affiliated organizations, or those of the publisher, the editors and the reviewers. Any product that may be evaluated in this article, or claim that may be made by its manufacturer, is not guaranteed or endorsed by the publisher.
