# Supplementary material for: Future-oriented cognition: links to mental health problems and mental wellbeing in preschool-aged and primary-school-aged children
Source: Front Psychol. 2023 Sep 27;14:1211986. doi: 10.3389/fpsyg.2023.1211986 (PMC10565826; doi:10.3389/fpsyg.2023.1211986)
Supplement: Supplementary file 1 [file Data_Sheet_1.docx]

| *Table 1.* Bivariate correlations for age, gender, CFQ and all subscales, SDQ and all subscales, PMH, and PLOT and all subscales. | | | | | | | | | | | | | | | | |
| --- | --- | --- | --- | --- | --- | --- | --- | --- | --- | --- | --- | --- | --- | --- | --- | --- |
|  | Age | Gender | CFTQ Total-score | SDQ Total-score | PMH Score | PLOT Total-score | CFTQ EFT Score | CFTQ Delay of Gratification Score | CFTQ Saving Score | CFTQ Planning Score | CFTQ Prospective Memory Score | SDQ Hyper-activity Score | SDQ Emotional Problem Score | SDQ Conduct Problem Score | PLOT Optimism Score | PLOT Pessimism Score |
| Age | 1 |  |  |  |  |  |  |  |  |  |  |  |  |  |  |  |
| Gender | -.064 | 1 |  |  |  |  |  |  |  |  |  |  |  |  |  |  |
| CFTQ Total Score | .441^**^ | -.166^*^ | 1 |  |  |  |  |  |  |  |  |  |  |  |  |  |
| SDQ Tota Score | -.083 | .,074 | -.323^**^ | 1 |  |  |  |  |  |  |  |  |  |  |  |  |
| PMH Total Score | .107 | -.057 | .244^**^ | -.653^**^ | 1 |  |  |  |  |  |  |  |  |  |  |  |
| PLOT Total Score | -.144 | -.044 | .150^*^ | -.507^**^ | .601^**^ | 1 |  |  |  |  |  |  |  |  |  |  |
| CFTQ-EFT | .352^**^ | -.133 | .803^**^ | -.213^**^ | .216^**^ | .176^*^ | 1 |  |  |  |  |  |  |  |  |  |
| CFTQ-DGI | .290^**^ | -.061 | .666^**^ | -.304^**^ | .204^**^ | .150^*^ | .357^**^ | 1 |  |  |  |  |  |  |  |  |
| CFTQ-Saving | .455^**^ | -.105 | .835^**^ | -.269^**^ | .227^**^ | .097 | .588^**^ | .487^**^ | 1 |  |  |  |  |  |  |  |
| CFTQ-Planning | .336^**^ | -.218^**^ | .861^**^ | -.292^**^ | .174^*^ | .111 | .651^**^ | .440^**^ | .632^**^ | 1 |  |  |  |  |  |  |
| CFTQ-PMI | .344^**^ | -.114 | .856^**^ | -.196^**^ | .158^*^ | .084 | .669^**^ | .379^**^ | .698^**^ | .730^**^ | 1 |  |  |  |  |  |
| SDQ-Hyperactivity | -.057 | .119 | -.449^**^ | .706^**^ | -.339^**^ | -.292^**^ | -.315^**^ | -.320^**^ | -.374^**^ | -.429^**^ | -.354^**^ | 1 |  |  |  |  |
| SDQ-Emotional  Problems | .061 | -.037 | -.034 | .670^**^ | -.558^**^ | -.485^**^ | -.008 | -.107 | .004 | -.010 | .020 | .204^**^ | 1 |  |  |  |
| SDQ-Conduct Problems | -.228^**^ | .123 | -.304^**^ | .653^**^ | -.416^**^ | -.231^**^ | -.205^**^ | -.255^**^ | -.302^**^ | -.237^**^ | -.223^**^ | .398^**^ | .176^*^ | 1 |  |  |
| PLOT-Optimism | -.117 | -.088 | .160^*^ | -.349^**^ | .438^**^ | .849^**^ | .196^**^ | .119 | .095 | .133 | .131 | -.217^**^ | -.305^**^ | -.190^**^ | 1 |  |
| PLOT-Pessimsm | .121 | -.021 | -.083 | .498^**^ | -.565^**^ | -.805^**^ | -.090 | -.130 | -.063 | -.046 | .000 | .269^**^ | .511^**^ | .192^**^ | -.370^**^ | 1 |
| *Note.* *N* = 191 (CFTQ, SDQ, and PMH) and *N* = 186 (PLOT). CFTQ (Children’s Future Thinking Questionnaire and subscales), SDQ (Strengths and Difficulties Questionnaire and Subscales), PMH (Positive Mental Health Scale), PLOT (Parent-rated Life Orientation Test of Children).  The correlation is significant at the 0.01 level (2-sided)**. The correlation is significant at the 0.05 level (2-sided)*. | | | | | | | | | | | | | | | | |

| *Table 2.* Regression analyses and 95% Confidence Interval (CI) predicting CFTQ by SDQ (and subscales) and accounting for age and gender (H_1a_). And interaction of age x SDQ and subscales predicting CFTQ (H_1b_). | | | | | | |
| --- | --- | --- | --- | --- | --- | --- |
|  |  | *b^a^* | *SE*_b_*^a^* | ß | *t* | *p* |
|  | **Regression 1: SDQ Total Score** | | | | | |
|  | Age | .184  [.119, .249] | .032 | .400 | 6.51 | ≤ .001 |
|  | Gender | -.143  [-.277, -.018] | .066 | -.127 | -2.10 | .023 |
|  |  |  |  |  |  |  |
|  | Total Score | -.032  [-.049, -.014] | .009 | -.233 | -3.64 | ≤ .001 |
|  | Age x Total Score | .011  [-.003, .026] | .008 | .108 | 1.68 | .094 |
|  |  |  |  |  |  |  |
|  | **Regression 2 : SDQ Subscales** | |  |  |  |  |
|  | Age | .180  [.121, .238] | .030 | .392 | 6.58 | ≤ .001 |
|  | Gender | -.109  [-.232, .017] | .062 | -.097 | -1.65 | .101 |
|  |  |  |  |  |  |  |
|  | Hyperactivity | -.113  [-.146, -.072] | .018 | -.410 | -6.27 | ≤ .001 |
|  | Age x Hyperactivity | .001  [-.029, .033] | .015 | .005 | .07 | .943 |
|  |  |  |  |  |  |  |
|  | Emotional Problem | .000  [-.036, .033] | .018 | .001 | .01 | .991 |
|  | Age x Emotional Problem | .002  [-.028, .033] | .015 | .007 | .12 | .908 |
|  |  |  |  |  |  |  |
|  | Conduct Problem | -.017  [-.067, .032] | .025 | -.046 | -.71 | .481 |
|  | Age x Conduct Problem | .018  [-.027, .059] | .021 | .066 | .97 | .332 |
|  | *Note.* *N* = 191. CFTQ (Children’s Future Thinking Questionnaire), SDQ (Strengths and Difficulties Questionnaire and subscales).  Regression 1: R² =.27; Regression 2: R² .39  ^a^Confidence intervals [CI] and standard errors (SE_b_) by BCa bootstrapping with 1000 BCa samples. | | | | | |

| *Table 3.* Regression analyses and 95% Confidence Interval (CI) predicting CFTQ by PMH and PLOT and accounting for age and gender (H_2a_). And interaction of age x PMH / PLOT predicting CFTQ (H_2b_). | | | | | |
| --- | --- | --- | --- | --- | --- |
|  | *b^a^* | *SE*_b_*^a^* | ß | *t* | *p* |
| **Regression 1: PMH Total Score** | |  |  |  |  |
| Age | .202  [.137, .263] | .033 | .443 | 6.65 | ≤ .001 |
| Gender | -.144  [-.278, -.010] | .072 | -.126 | -1.96 | .052 |
| Total Score | .169  [-.087, .462] | .142 | .100 | 1.22 | .225 |
| Age x Total Score | -.083  [-.340, 172] | .129 | -.067 | -.76 | .451 |
|  |  |  |  |  |  |
| **PLOT Total Score** |  |  |  |  |  |
| Total Score | .179  [-.034, .376] | .106 | .147 | 1.78 | .077 |
| Age x Total Score | .098  [-.121, .335] | .114 | .101 | 1.12 | .264 |
|  |  |  |  |  |  |
| **Regression 2: PLOT Subscales** | |  |  |  |  |
| Optimism | .136  [9.52, .257] | .066 | .142 | 1.95 | .053 |
| Age x Optimism | -.068  [-.163, .049] | .054 | -.091 | -1.29 | .199 |
|  |  |  |  |  |  |
| Pessimism | -.023  [-.203, .159] | .091 | -.022 | -.27 | .786 |
| Age x Pessimism | -.119  [-.250, .007] | .064 | -.132 | 2.03 | .044 |
| *Note.* *N* = 185. CFTQ (Children’s Future Thinking Questionnaire), PMH Total score (Positive Mental Health Scale), PLOT Total Score and subscales (Parent-rated Life Orientation Test of Children).  Regression 1: R² = .27; Regression 2: R² = .28  ^a^Confidence intervals [CI] and standard errors (SE_b_) by BCa bootstrapping with 1000 BCa samples. | | | | | |

| *Table 4.* Results of explorative regression analyses for the relation between SDQ Total Score and CFTQ-EFT. And interaction of age x SDQ Total Score predicting CFTQ-EFT. | | | | | | |
| --- | --- | --- | --- | --- | --- | --- |
|  |  | *b^a^* | *SE*_b_*^a^* | ß | *t* | *p* |
|  | **SDQ Total Score** | | | | | |
|  | Age | .181  [.106, .259] | .040 | .321 | 4.79 | ≤ .001 |
|  | Gender | -.146  [-.336, -.023] | .092 | -.106 | -1.57 | .118 |
|  |  |  |  |  |  |  |
|  | CFTQ-EFT | -.028  [-.048, -.008] | .010 | -.188 | -2.79 | .006 |
|  | Age x CFTQ-EFT | .011  [-.006, .027] | .011 | .084 | 1.22 | .226 |
|  | *Note.* *N* = 191. CFTQ-EFT (Children’s Future Thinking Questionnaire - Episodic Foresight subscale), SDQ Total Score (Strengths and Difficulties Questionnaire)  R² = .16.  ^a^Confidence intervals [CI] and standard errors (SE_b_) by BCa bootstrapping with 1000 BCa samples. | | | | | |

| *Table 5.* Results of explorative regression analyses for the relation between SDQ Total Score and CFTQ-DGI. And interaction of age x SDQ Total Score predicting CFTQ-DGI. | | | | | | |
| --- | --- | --- | --- | --- | --- | --- |
|  |  | *b^a^* | *SE*_b_*^a^* | ß | *t* | *p* |
|  | **SDQ Total Score** | | | | | |
|  | Age | .152  [.076, .237] | .040 | .266 | 3.88 | ≤ .001 |
|  | Gender | -.068  [-.251, .119] | .094 | -.049 | -.72 | .475 |
|  |  |  |  |  |  |  |
|  | CFTQ-DGI | -.045  [-.065, -.025] | .010 | -.294 | -4.38 | ≤ .001 |
|  | Age x CFTQ-DGI | .014  [-.004, .032] | .009 | .109 | 1.58 | .115 |
|  | *Note.* *N* = 191. CFTQ-DGI (Children’s Future Thinking Questionnaire - Delay of Gratification subscale), SDQ Total Score (Strengths and Difficulties Questionnaire)  R² = .16.  ^a^Confidence intervals [CI] and standard errors (SE_b_) by BCa bootstrapping with 1000 BCa samples. | | | | | |

| *Table 6.* Results of explorative regression analyses for the relation between SDQ Total Score and CFTQ-PMI. And interaction of age x SDQ Total Score predicting CFTQ-PMI. | | | | | | |
| --- | --- | --- | --- | --- | --- | --- |
|  |  | *b^a^* | *SE*_b_*^a^* | ß | *t* | *p* |
|  | **SDQ Total Score** | | | | | |
|  | Age | .186  [.095, .268] | .045 | .318 | 4.63 | ≤ .001 |
|  | Gender | -.160  [-.348, .047] | .101 | -.131 | -1.91 | .058 |
|  |  |  |  |  |  |  |
|  | CFTQ-PMI | -.029  [-.051, -.008] | .011 | -.189 | -2.80 | .006 |
|  | Age x CFTQ-PMI | .008  [-.012, .027] | .010 | .061 | .88 | .378 |
|  | *Note.* *N* = 191. CFTQ-PMI (Children’s Future Thinking Questionnaire – Prospective Memory subscale), SDQ Total Score (Strengths and Difficulties Questionnaire)  R² = .15.  ^a^Confidence intervals [CI] and standard errors (SE_b_) by BCa bootstrapping with 1000 BCa samples. | | | | | |

| *Table 7.* Results of explorative regression analyses for the relation between SDQ Total Score and CFTQ-Saving. And interaction of age x SDQ Total Score predicting CFTQ-Saving. | | | | | | |
| --- | --- | --- | --- | --- | --- | --- |
|  |  | *b^a^* | *SE*_b_*^a^* | ß | *t* | *p* |
|  | **SDQ Total Score** | | | | | |
|  | Age | .204  [.136, .270] | .034 | .418 | 6.42 | ≤ .001 |
|  | Gender | -.097  [-.247, .044] | .072 | -.081 | -1.26 | .209 |
|  |  |  |  |  |  |  |
|  | CFTQ-Saving | -.032  [-.049, -.016] | .009 | -.247 | -3.88 | ≤ .001 |
|  | Age x CFTQ-Saving | .005  [-.010, .021] | .008 | .047 | .71 | .477 |
|  | *Note.* *N* = 191. CFTQ-Saving (Children’s Future Thinking Questionnaire – Saving subscale), SDQ Total Score (Strengths and Difficulties Questionnaire)  R² = .23.  ^a^Confidence intervals [CI] and standard errors (SE_b_) by BCa bootstrapping with 1000 BCa samples. | | | | | |

| *Table 8.* Results of explorative regression analyses for the relation between SDQ Total Score and CFTQ-Planning. And interaction of age x SDQ Total Score predicting CFTQ-Planning. | | | | | | |
| --- | --- | --- | --- | --- | --- | --- |
|  |  | *b^a^* | *SE*_b_*^a^* | ß | *t* | *p* |
|  | **SDQ Total Score** | | | | | |
|  | Age | .178  [.077, .266] | .049 | .297 | 4.49 | ≤ .001 |
|  | Gender | -.289  [-.468, -.099] | .095 | -.198 | -3.01 | .003 |
|  |  |  |  |  |  |  |
|  | CFTQ-Planning | -.042  [-.064, -.023] | .010 | -.263 | -4.04 | ≤ .001 |
|  | Age x CFTQ-Planning | .014  [-.005, .035] | .010 | .102 | 1.52 | .132 |
|  | *Note.* *N* = 191. CFTQ-Planning (Children’s Future Thinking Questionnaire – Planning subscale), SDQ Total Score (Strengths and Difficulties Questionnaire)  R² = .23.  ^a^Confidence intervals [CI] and standard errors (SE_b_) by BCa bootstrapping with 1000 BCa samples. | | | | | |

| *Table 9.* Results of explorative regression analyses for the relation between SDQ subscales and CFTQ-EFT. And interaction of age x SDQ subscales predicting CFTQ-EFT. | | | | | | |
| --- | --- | --- | --- | --- | --- | --- |
|  |  | *b^a^* | *SE*_b_*^a^* | ß | *t* | *p* |
|  |  | | | | | |
|  | Age | .173  [.094, .252] | .041 | .307 | 4.51 | ≤ .001 |
|  | Gender | -.118  [-.295, .071] | .094 | -.085 | -1.27 | .205 |
|  | **Hyperactivity** |  |  |  |  |  |
|  | CFTQ-EFT | -.098  [-.147, -.044] | .026 | -.290 | -3.99 | ≤ .001 |
|  | Age x CFTQ-EFT | .014  [-.030, .051] | .020 | .052 | .71 | .476 |
|  | **Emotional Problems** |  |  |  |  |  |
|  | CFTQ-EFT | .006  [-.038, .056] | .024 | .016 | .23 | .815 |
|  | Age x CFTQ-EFT | -.008  [-.048, .031] | .021 | -.026 | -.38 | .706 |
|  | **Conduct Problems** |  |  |  |  |  |
|  | CFTQ-EFT | -.007  [-.079, .057] | .035 | -.016 | -.21 | .831 |
|  | Age x CFTQ-EFT | .016  [-.036, .066] | .026 | .049 | .64 | .520 |
|  | *Note.* *N* = 191. CFTQ-EFT (Children’s Future Thinking Questionnaire – Episodic Foresight subscale), SDQ subscales (Strengths and Difficulties Questionnaire - Hyperactivity, Emotional Problems, Conduct Problems subscales)  R² = .22.  ^a^Confidence intervals [CI] and standard errors (SE_b_) by BCa bootstrapping with 1000 BCa samples. | | | | | |

| *Table 10.* Results of explorative regression analyses for the relation between SDQ subscales and CFTQ-DGI. And interaction of age x SDQ subscales predicting CFTQ-DGI. | | | | | | |
| --- | --- | --- | --- | --- | --- | --- |
|  |  | *b^a^* | *SE*_b_*^a^* | ß | *t* | *p* |
|  |  | | | | | |
|  | Age | .150  [.068, .232] | .042 | .262 | 3.83 | ≤ .001 |
|  | Gender | .002  [-.174, .183] | .089 | .001 | .017 | .987 |
|  | **Hyperactivity** |  |  |  |  |  |
|  | CFTQ-DGI | -.091  [-.143, -.040] | .026 | -.264 | -3.58 | ≤ .001 |
|  | Age x CFTQ-DGI | -.030  [-.070, .008] | .020 | -.114 | -1.55 | .122 |
|  | **Emotional Problems** |  |  |  |  |  |
|  | CFTQ-DGI | -.029  [-.090, .020] | .028 | -.075 | -1.09 | .276 |
|  | Age x CFTQ-DGI | .023  [-.020, .070] | .023 | .075 | 1.07 | .286 |
|  | **Conduct Problems** |  |  |  |  |  |
|  | CFTQ- DGI | -.036  [-.109, .034] | .036 | -.077 | -1.03 | .305 |
|  | Age x CFTQ- DGI | .044  [-.008, .095] | .027 | .133 | 1.74 | .083 |
|  | *Note.* *N* = 191. CFTQ-DGI (Children’s Future Thinking Questionnaire – Delay of Gratification subscale), SDQ subscales (Strengths and Difficulties Questionnaire - Hyperactivity, Emotional Problems, Conduct Problems subscales)  R² = .22.  ^a^Confidence intervals [CI] and standard errors (SE_b_) by BCa bootstrapping with 1000 BCa samples. | | | | | |

| *Table 11.* Results of explorative regression analyses for the relation between SDQ subscales and CFTQ-PMI. And interaction of age x SDQ subscales predicting CFTQ-PMI. | | | | | | |
| --- | --- | --- | --- | --- | --- | --- |
|  |  | *b^a^* | *SE*_b_*^a^* | ß | *t* | *p* |
|  |  | | | | | |
|  | Age | .176  [.089, .266] | .044 | .301 | 4.50 | ≤ .001 |
|  | Gender | -.114  [-.288, .092] | .093 | -.080 | -1.21 | .227 |
|  | **Hyperactivity** |  |  |  |  |  |
|  | CFTQ-PMI | -.118  [-.162, -.068] | .024 | -.337 | -4.71 | ≤ .001 |
|  | Age x CFTQ-PMI | .026  [-.019, .065] | .021 | .095 | 1.33 | .185 |
|  | **Emotional Problems** |  |  |  |  |  |
|  | CFTQ-PMI | .014  [-.035, .062] | .024 | .035 | .52 | .602 |
|  | Age x CFTQ-PMI | -.008  [-.053, .041] | .024 | -.025 | -.37 | .716 |
|  | **Conduct Problems** |  |  |  |  |  |
|  | CFTQ-PMI | -.010  [-.085, .062] | .038 | -.022 | -.30 | .767 |
|  | Age x CFTQ-PMI | -.017  [-.070, .037] | .028 | -.050 | -.66 | .507 |
|  | *Note.* *N* = 191. CFTQ-PMI (Children’s Future Thinking Questionnaire – Prospective Memory subscale), SDQ subscales (Strengths and Difficulties Questionnaire - Hyperactivity, Emotional Problems, Conduct Problems subscales)  R² = .25.  ^a^Confidence intervals [CI] and standard errors (SE_b_) by BCa bootstrapping with 1000 BCa samples. | | | | | |

| *Table 12.* Results of explorative regression analyses for the relation between SDQ subscales and CFTQ-Saving. And interaction of age x SDQ subscales predicting CFTQ-Saving. | | | | | | |
| --- | --- | --- | --- | --- | --- | --- |
|  |  | *b^a^* | *SE*_b_*^a^* | ß | *t* | *p* |
|  |  | | | | | |
|  | Age | .196  [.130, .265] | .034 | -.401 | -6.33 | ≤ .001 |
|  | Gender | -.039  [-.181, .102] | .070 | -.033 | -.52 | .602 |
|  | **Hyperactivity** |  |  |  |  |  |
|  | CFTQ-Saving | -.094  [-.130, -.052] | .020 | -.320 | -4.74 | ≤ .001 |
|  | Age x CFTQ-Saving | -.013  [-.049, .024] | .018 | -.056 | -.82 | .411 |
|  | **Emotional Problems** |  |  |  |  |  |
|  | CFTQ-Saving | .006  [-.035, .047] | .021 | .019 | .31 | .761 |
|  | Age x CFTQ-Saving | .009  [-.026, .042] | .017 | .033 | .52 | .607 |
|  | **Conduct Problems** |  |  |  |  |  |
|  | CFTQ-Saving | -.033  [-.092, .021] | .029 | -.084 | -1.22 | .223 |
|  | Age x CFTQ-Saving | -.005  [-.045, .041] | .022 | -.019 | -.27 | .784 |
|  | *Note.* *N* = 191. CFTQ-Saving (Children’s Future Thinking Questionnaire – Saving subscale), SDQ subscales (Strengths and Difficulties Questionnaire - Hyperactivity, Emotional Problems, Conduct Problems subscales)  R² = .33.  ^a^Confidence intervals [CI] and standard errors (SE_b_) by BCa bootstrapping with 1000 BCa samples. | | | | | |

| *Table 13.* Results of explorative regression analyses for the relation between SDQ subscales and CFTQ-Planning. And interaction of age x SDQ subscales predicting CFTQ-Planning. | | | | | | |
| --- | --- | --- | --- | --- | --- | --- |
|  |  | *b^a^* | *SE*_b_*^a^* | ß | *t* | *p* |
|  |  | | | | | |
|  | Age | .171  [.075, .259] | .047 | .286 | 4.48 | ≤ .001 |
|  | Gender | -.228  [-.405, -.071] | .081 | -.156 | -2.48 | .014 |
|  | **Hyperactivity** |  |  |  |  |  |
|  | CFTQ-Planning | -.143  [-.190, -.087] | .027 | -.401 | -5.87 | ≤ .001 |
|  | Age x CFTQ-Planning | .009  [-.038, .055] | .023 | .032 | .47 | .640 |
|  | **Emotional Problems** |  |  |  |  |  |
|  | CFTQ-Planning | .011  [-.039, .056] | .024 | .26 | .42 | .678 |
|  | Age x CFTQ-Planning | -.014  [-.058, .031] | .022 | -.042 | -.65 | .517 |
|  | **Conduct Problems** |  |  |  |  |  |
|  | CFTQ-Planning | -.001  [-.073, .069] | .036 | -.001 | -.02 | .987 |
|  | Age x CFTQ-Planning | .021  [-.037, .083] | .031 | .060 | .84 | .404 |
|  | *Note.* *N* = 191. CFTQ-Planning (Children’s Future Thinking Questionnaire – Planning subscale), SDQ subscales (Strengths and Difficulties Questionnaire - Hyperactivity, Emotional Problems, Conduct Problems subscales)  R² = .31.  ^a^Confidence intervals [CI] and standard errors (SE_b_) by BCa bootstrapping with 1000 BCa samples. | | | | | |

| *Table 14.* Results of explorative regression analyses for the relation between PMH Total Score, PLOT Total Score and CFTQ-EFT. And interaction of age x PMH Total Score / PLOT Total Score predicting CFTQ-EFT. | | | | | | |
| --- | --- | --- | --- | --- | --- | --- |
|  |  | *b^a^* | *SE*_b_*^a^* | ß | *t* | *p* |
|  |  | | | | | |
|  | Age | .206  [.124, .284] | .042 | .364 | 5.09 | ≤ .001 |
|  | Gender | -.136  [-.330, .045] | .098 | -.096 | -1.41 | .160 |
|  | **PMH Total Score** |  |  |  |  |  |
|  | CFTQ-EFT | .120  [-.189, .437] | .159 | .057 | .66 | .510 |
|  | Age x CFTQ-EFT | -.020  [-.325, .232] | .139 | -.013 | -.14 | .892 |
|  | **PLOT Total Score** |  |  |  |  |  |
|  | CFTQ-EFT | .278  [-.006, .549] | .139 | .185 | 2.08 | .039 |
|  | Age x CFTQ-EFT | .040  [-.208, .299] | .130 | .033 | .35 | .725 |
|  | *Note.* *N* = 185. CFTQ-EFT (Children’s Future Thinking Questionnaire – Episodic Foresight subscale), PMH Total Score (Positive Mental Health Scale), PLOT Total Score (Parent-rated Life Orientation Test of Children).  R² = .19.  ^a^Confidence intervals [CI] and standard errors (SE_b_) by BCa bootstrapping with 1000 BCa samples. | | | | | |

| *Table 15.* Results of explorative regression analyses for the relation between PMH Total Score, PLOT Total Score and CFTQ-DGI. And interaction of age x PMH Total Score / PLOT Total Score predicting CFTQ-DGI. | | | | | | |
| --- | --- | --- | --- | --- | --- | --- |
|  |  | *b^a^* | *SE*_b_*^a^* | ß | *t* | *p* |
|  |  | | | | | |
|  | Age | .167  [.076, .254] | .045 | .292 | 3.95 | ≤ .001 |
|  | Gender | -.043  [-.255, .174] | .108 | -.030 | -0.43 | .672 |
|  | **PMH Total Score** |  |  |  |  |  |
|  | CFTQ-DGI | .183  [-.167, .545] | .178 | .087 | .95 | .338 |
|  | Age x CFTQ- DGI | -.082  [-.389, .192] | .146 | -.053 | -.55 | .585 |
|  | **PLOT Total Score** |  |  |  |  |  |
|  | CFTQ-DGI | .209  [-.044, .462] | .134 | .137 | 1.49 | .138 |
|  | Age x CFTQ- DGI | .038  [-.201, .289] | .127 | .031 | .32 | .752 |
|  | *Note.* *N* = 185. CFTQ-DGI (Children’s Future Thinking Questionnaire – Delay of Gratification subscale), PMH Total Score (Positive Mental Health Scale), PLOT Total Score (Parent-rated Life Orientation Test of Children).  R² = .13.  ^a^Confidence intervals [CI] and standard errors (SE_b_) by BCa bootstrapping with 1000 BCa samples. | | | | | |

| *Table 16.* Results of explorative regression analyses for the relation between PMH Total Score, PLOT Total Score and CFTQ-PMI. And interaction of age x PMH Total Score / PLOT Total Score predicting CFTQ-PMI. | | | | | | |
| --- | --- | --- | --- | --- | --- | --- |
|  |  | *b^a^* | *SE*_b_*^a^* | ß | *t* | *p* |
|  |  | | | | | |
|  | Age | .199  [.108, .279] | .047 | .343 | 4.69 | ≤ .001 |
|  | Gender | -.117  [-.306, .104] | .104 | -.081 | -1.16 | .248 |
|  | **PMH Total Score** |  |  |  |  |  |
|  | CFTQ-PMI | .133  [-.226, .515] | .189 | .062 | .69 | .487 |
|  | Age x CFTQ- PMI | -.003  [-.403, .349] | .187 | -.002 | -.02 | .585 |
|  | **PLOT Total Score** |  |  |  |  |  |
|  | CFTQ-PMI | .131  [-.171, .383] | .141 | .085 | .93 | .354 |
|  | Age x CFTQ- PMI | .050  [-.231, .356] | .149 | .040 | .41 | .679 |
|  | *Note.* *N* = 185. CFTQ-PMI (Children’s Future Thinking Questionnaire – Prospective Memory subscale), PMH Total Score (Positive Mental Health Scale), PLOT Total Score (Parent-rated Life Orientation Test of Children).  R² = .15.  ^a^Confidence intervals [CI] and standard errors (SE_b_) by BCa bootstrapping with 1000 BCa samples. | | | | | |

| *Table 17.* Results of explorative regression analyses for the relation between PMH Total Score, PLOT Total Score and CFTQ-Saving. And interaction of age x PMH Total Score / PLOT Total Score predicting CFTQ-Saving. | | | | | | |
| --- | --- | --- | --- | --- | --- | --- |
|  |  | *b^a^* | *SE*_b_*^a^* | ß | *t* | *p* |
|  |  | | | | | |
|  | Age | .210  [.143, .287] | .037 | .436 | 6.39 | ≤ .001 |
|  | Gender | -.065  [-.207, .94] | .075 | -.054 | -.84 | .405 |
|  | **PMH Total Score** |  |  |  |  |  |
|  | CFTQ-Saving | .235  [-.032, .569] | .150 | .132 | 1.59 | .115 |
|  | Age x CFTQ- Saving | -.094  [-.342, .172] | .128 | -.072 | -.81 | .422 |
|  | **PLOT Total Score** |  |  |  |  |  |
|  | CFTQ-Saving | .070  [-.161, .257] | .110 | .054 | .64 | .522 |
|  | Age x CFTQ- Saving | .163  [-.053, .386] | .109 | .158 | 1.75 | .081 |
|  | *Note.* *N* = 185. CFTQ-Saving (Children’s Future Thinking Questionnaire – Saving subscale), PMH Total Score (Positive Mental Health Scale), PLOT Total Score (Parent-rated Life Orientation Test of Children).  R² = .26.  ^a^Confidence intervals [CI] and standard errors (SE_b_) by BCa bootstrapping with 1000 BCa samples. | | | | | |

| *Table 18.* Results of explorative regression analyses for the relation between PMH Total Score, PLOT Total Score and CFTQ-Planning. And interaction of age x PMH Total Score / PLOT Total Score predicting CFTQ-Planning. | | | | | | |
| --- | --- | --- | --- | --- | --- | --- |
|  |  | *b^a^* | *SE*_b_*^a^* | ß | *t* | *p* |
|  |  | | | | | |
|  | Age | .189  [.090, .279] | .049 | .318 | 4.44 | ≤ .001 |
|  | Gender | -.267  [-.496, -.057] | .104 | -.179 | -2.64 | .009 |
|  | **PMH Total Score** |  |  |  |  |  |
|  | CFTQ-Planning | .139  [-.194, .524] | .180 | .063 | .73 | .468 |
|  | Age x CFTQ- Planning | -.161  [-.514, .150] | .166 | -.100 | -1.08 | .284 |
|  | **PLOT Total Score** |  |  |  |  |  |
|  | CFTQ-Planning | .145  [-.142, .387] | .130 | .092 | 1.03 | .305 |
|  | Age x CFTQ- Planning | .188  [-.080, .478] | .137 | .148 | 1.57 | .119 |
|  | *Note.* *N* = 185. CFTQ-Planning (Children’s Future Thinking Questionnaire – Planning subscale), PMH Total Score (Positive Mental Health Scale), PLOT Total Score (Parent-rated Life Orientation Test of Children).  R² = .19.  ^a^Confidence intervals [CI] and standard errors (SE_b_) by BCa bootstrapping with 1000 BCa samples. | | | | | |

| *Table 19.* Results of explorative regression analyses for the relation between PLOT subscales and CFTQ-EFT. And interaction of age x PLOT Subscales predicting CFTQ-EFT. | | | | | | |
| --- | --- | --- | --- | --- | --- | --- |
|  |  | *b^a^* | *SE*_b_*^a^* | ß | *t* | *p* |
|  |  | | | | | |
|  | Age | .202  [.120, .286] | .041 | .357 | 5.02 | ≤ .001 |
|  | Gender | -.135  [-.328, -.056] | .099 | -.095 | -1.40 | .164 |
|  | **PLOT-Optimism** |  |  |  |  |  |
|  | CFTQ-EFT | .226  [.041, .416] | .092 | .192 | 2.47 | .015 |
|  | Age x CFTQ- EFT | -.053  [-.181, .107] | .073 | -.057 | -.72 | .475 |
|  | **PLOT-Pessimism** |  |  |  |  |  |
|  | CFTQ-EFT | -.015  [-.209, .214] | .108 | -.012 | -.14 | .891 |
|  | Age x CFTQ- EFT | -.144  [-.368, .087] | .113 | -.129 | -1.43 | .154 |
|  | *Note.* *N* = 185. CFTQ-EFT (Children’s Future Thinking Questionnaire – Episodic Foresight subscale), PLOT Subscales (Parent-rated Life Orientation Test of Children, Optimism Subscale, Pessimism Subscale).  R² = .21.  ^a^Confidence intervals [CI] and standard errors (SE_b_) by BCa bootstrapping with 1000 BCa samples. | | | | | |

| *Table 20.* Results of explorative regression analyses for the relation between PLOT subscales and CFTQ-DGI. And interaction of age x PLOT Subscales predicting CFTQ-DGI. | | | | | | |
| --- | --- | --- | --- | --- | --- | --- |
|  |  | *b^a^* | *SE*_b_*^a^* | ß | *t* | *p* |
|  |  | | | | | |
|  | Age | .167  [.070, .257] | .048 | .291 | 3.91 | ≤ .001 |
|  | Gender | -.051  [-.253, .142] | .102 | -.035 | -.49 | .619 |
|  | **PLOT-Optimism** |  |  |  |  |  |
|  | CFTQ-DGI | .099  [-.112, .294] | .103 | .083 | .89 | .374 |
|  | Age x CFTQ- DGI | -.023  [-.182, .178] | .086 | -.025 | -.29 | .767 |
|  | **PLOT-Pessimism** |  |  |  |  |  |
|  | CFTQ-DGI | -.114  [-.348, .145] | .122 | -.085 | -.96 | .339 |
|  | Age x CFTQ- DGI | -.093  [-.292, .157] | .118 | -.082 | -.87 | .384 |
|  | *Note.* *N* = 185. CFTQ-DGI (Children’s Future Thinking Questionnaire –Delay of Gratification subscale), PLOT Subscales (Parent-rated Life Orientation Test of Children, Optimism Subscale, Pessimism Subscale).  R² = .13.  ^a^Confidence intervals [CI] and standard errors (SE_b_) by BCa bootstrapping with 1000 BCa samples. | | | | | |

| *Table 21.* Results of explorative regression analyses for the relation between PLOT subscales and CFTQ-PMI. And interaction of age x PLOT Subscales predicting CFTQ-PMI. | | | | | | |
| --- | --- | --- | --- | --- | --- | --- |
|  |  | *b^a^* | *SE*_b_*^a^* | ß | *t* | *p* |
|  |  | | | | | |
|  | Age | .194  [.109, .285] | .046 | .334 | 4.62 | ≤ .001 |
|  | Gender | -.117  [-.314, .062] | .096 | -.081 | -1.16 | .245 |
|  | **PLOT-Optimism** |  |  |  |  |  |
|  | CFTQ-PMI | .186  [-.035, .361] | .101 | .154 | 1.95 | .052 |
|  | Age x CFTQ- PMI | -.085  [-.243, .124] | .090 | -.089 | -1.10 | .273 |
|  | **PLOT-Pessimism** |  |  |  |  |  |
|  | CFTQ-PMI | .106  [-.118, .328] | .109 | .078 | .91 | .365 |
|  | Age x CFTQ- PMI | -.210  [-.428, .030] | .114 | -.184 | -2.01 | .045 |
|  | *Note.* *N* = 185. CFTQ-PMI (Children’s Future Thinking Questionnaire – Prospective Memory subscale), PLOT Subscales (Parent-rated Life Orientation Test of Children, Optimism Subscale, Pessimism Subscale).  R² = .18.  ^a^Confidence intervals [CI] and standard errors (SE_b_) by BCa bootstrapping with 1000 BCa samples. | | | | | |

| *Table 22.* Results of explorative regression analyses for the relation between PLOT subscales and CFTQ-Saving. And interaction of age x PLOT Subscales predicting CFTQ-Saving. | | | | | | |
| --- | --- | --- | --- | --- | --- | --- |
|  |  | *b^a^* | *SE*_b_*^a^* | ß | *t* | *p* |
|  |  | | | | | |
|  | Age | .207  [.139, .271] | .034 | .430 | 6.36 | ≤ .001 |
|  | Gender | -.075  [-.216, .076] | .075 | -.062 | -.96 | .339 |
|  | **PLOT-Optimism** |  |  |  |  |  |
|  | CFTQ-Saving | .070  [-.085, .209] | .072 | .069 | .94 | .349 |
|  | Age x CFTQ- Saving | -.007  [-.125, .115] | .060 | -.009 | -.12 | .903 |
|  | **PLOT-Pessimism** |  |  |  |  |  |
|  | CFTQ-Saving | .013  [-.209, .214] | .108 | -.012 | -.14 | .891 |
|  | Age x CFTQ- Saving | -.144  [-.146, .166] | .081 | -.246 | -2.88 | .004 |
|  | *Note.* *N* = 185. CFTQ-Saving (Children’s Future Thinking Questionnaire – Saving subscale), PLOT Subscales (Parent-rated Life Orientation Test of Children, Optimism Subscale, Pessimism Subscale).  R² = .28.  ^a^Confidence intervals [CI] and standard errors (SE_b_) by BCa bootstrapping with 1000 BCa samples. | | | | | |

| *Table 23.* Results of explorative regression analyses for the relation between PLOT subscales and CFTQ-Planning. And interaction of age x PLOT Subscales predicting CFTQ-Planning. | | | | | | |
| --- | --- | --- | --- | --- | --- | --- |
|  |  | *b^a^* | *SE*_b_*^a^* | ß | *t* | *p* |
|  |  | | | | | |
|  | Age | .184  [.092, .277] | .047 | .310 | 4.40 | ≤ .001 |
|  | Gender | -.280  [-.459, -.080] | .098 | -.187 | -2.79 | .006 |
|  | **PLOT-Optimism** |  |  |  |  |  |
|  | CFTQ-Planning | .147  [-.045, .239] | .085 | .118 | 1.54 | .125 |
|  | Age x CFTQ- Planning | -.047  [-.203, .133] | .082 | -.048 | -.60 | .456 |
|  | **PLOT-Pessimism** |  |  |  |  |  |
|  | CFTQ-Planning | .031  [-.186, .275] | .111 | .022 | .266 | .790 |
|  | Age x CFTQ- Planning | -.047  [-.543, -.080] | .118 | -.286 | -3.22 | .002 |
|  | *Note.* *N* = 185. CFTQ-Saving (Children’s Future Thinking Questionnaire – Planning subscale), PLOT Subscales (Parent-rated Life Orientation Test of Children, Optimism Subscale, Pessimism Subscale).  R² = .23.  ^a^Confidence intervals [CI] and standard errors (SE_b_) by BCa bootstrapping with 1000 BCa samples. | | | | | |
